# Supplementary material for: Genetic Evidence for a Mixed Composition of the Genus Myoxocephalus (Cottoidei: Cottidae) Necessitates Generic Realignment
Source: Genes (Basel). 2020 Sep 11;11(9):1071. doi: 10.3390/genes11091071 (PMC7564937; doi:10.3390/genes11091071)
Supplement: Supplementary file 1 [file genes-11-01071-s001.zip › Table_S1_Sites_of_specimen_collection.doc]

**Table S1. Sites of specimen collection for the study**

| Genus | Species | Number | Date | Locality | N | E |
| --- | --- | --- | --- | --- | --- | --- |
| *Argyrocottus* | *zanderi* | AZA1 | 23.07.2017 | The Sea of Japan, Olga Bay | 43.71976 | 135.25538 |
| *Argyrocottus* | *zanderi* | AZA4 | 24.07.2017 | The Sea of Japan, Vladimir Bay | 43.87549 | 135.47141 |
| *Megalocottus* | *platycephalus* | MPL3, MPL5 | 26.07.2017 | The Sea of Japan, Djigit Bay, Kluchi River | 44.79675 | 136.39252 |
| *Microcottus* | *sellaris* | MSE1 | 19.01.2018 | The Sea of Japan, Gulf of Vladimir, South Bay | 43.882738 | 135.457252 |
| *Microcottus* | *sellaris* | MSE4 | 21.01.2018 | The Sea of Japan, Gulf of Vladimir, South Bay | 43.882738 | 135.457252 |
| *Myoxocephalus* | *quadricornis* | MT303953, MT303954 | 18.03.2017 | The White sea, Dvina Bay | 64.5553 | 40.1719 |
| *Myoxocephalus* | *brandtii* | MBR2, MBR3 | 23.07.2017 | The Sea of Japan, Olga Bay | 43.71976 | 135.25538 |
| *Myoxocephalus* | *jaok* | MJA4 | 07.08.2017 | The Sea of Japan, Sakhalin Island, near Alexandrovsk-Sakhalinsky | 50.89277 | 142.10077 |
| *Myoxocephalus* | *jaok* | MJA5 | 09.08.2017 | The Sea of Japan, De-Kastri Bay, northern part | 51.48547 | 140.82197 |
| *Myoxocephalus* | *polyacanthocephalus* | MPO5 | 30.07.2017 | The Sea of Japan, abeam Salt (Solyonoye) Lake | 47.17448 | 47.17448 |
| *Myoxocephalus* | *polyacanthocephalus* | MPO4 | 29.07.2017 | Sea of Japan, abeam Malaya Kema River | 45.39341 | 137.22861 |
| *Myoxocephalus* | *stelleri* | MST36 | 10.08.2017 | The Sea of Japan, De-Kastri Bay, Gulf Tigil | 51.4253 | 140.8157 |
| *Porocottus* | *allisi* | PAL3 | 26.07.2017 | The Sea of Japan, Djigit Bay, Kluchi River | 44.79677 | 136.39254 |
| *Porocottus* | *allisi* | PAL4 | 31.07.2017 | The Sea of Japan, Zolotoy Cape | 47.3128 | 138.9698 |
| *Porocottus* | *japonicus* | PJA3, PJA4 | 26.07.2017 | The Sea of Japan, Djigit Bay | 44.79676 | 136.39253 |
| *Porocottus* | *minutus* | MPI3, MPI5 | August 2017 | The Sea of Okhotsk, Odyan Bay, mouth of the Calcutta River | 59.14763 | 152.01344 |
