# Supplementary material for: Genetic Evidence for a Mixed Composition of the Genus Myoxocephalus (Cottoidei: Cottidae) Necessitates Generic Realignment
Source: Genes (Basel). 2020 Sep 11;11(9):1071. doi: 10.3390/genes11091071 (PMC7564937; doi:10.3390/genes11091071)
Supplement: Supplementary file 1 [file genes-11-01071-s001.zip › Table_S2_Species_names_and_GenBank_Accession_Numbers.doc]

Table S2. Species names and GenBank accession numbers

| Species | GenBank accession number | Reference |
| --- | --- | --- |
| *Clinocottus analis* | FJ848374 | Ramon et al. 2016 |
| *Comephorus baicalensis* | MF346886 | Sandel et al. 2019 |
| *Comephorus dybowskii* | MF346888 | Sandel et al. 2019 |
| *Cottiusculus nihonkaiensis* | MK224511 | Yang et al. 2019 |
| *Enophrys bison* | JQ354085 | Elz et al. 2014 |
| *Enophrys bison* | JQ354084 | Elz et al. 2014 |
| *Enophrys bison* | KP827347 | Buser et al. 2016 |
| *Enophrys diceraus* | HQ712362 | Mecklenburg et al. 2011 |
| *Enophrys diceraus* | HQ712363 | Mecklenburg et al. 2011 |
| *Enophrys diceraus* | HQ712364 | Mecklenburg et al. 2011 |
| *Enophrys diceraus* | KF039715 | Li et al. 2013 |
| *Enophrys lucasi* | KF929844 | Bentley et al. 2014 |
| *Enophrys lucasi* | KP827352 | Buser et al. 2016 |
| *Enophrys taurina* | KF918873 | Elz et al. 2014 |
| *Enophrys taurina* | KF918872 | Elz et al. 2014 |
| *Gymnocanthus herzensteini* | KX148474 | Kim et al. 2017 |
| *Gymnocanthus intermedius* | KX148473 | Kim et al. 2017 |
| *Hemilepidotus gilberti* | KX156764 | Song et al. 2017 |
| *Icelus spatula* | KT004432 | Swanburg et al. 2015 |
| *Megalocottus platycephalus* | JF278613 | Podlesnykh et al. 2016 |
| *Megalocottus platycephalus* | JF278614 | Podlesnykh et al. 2016 |
| *Megalocottus platycephalus* | JF278615 | Podlesnykh et al. 2016 |
| *Megalocottus platycephalus* | MH016199 | Radchenko et al. 2018 |
| *Megalocottus platycephalus* | MH016200 | Radchenko et al. 2018 |
| *Megalocottus platycephalus* | MH016201 | Radchenko et al. 2018 |
| *Megalocottus platycephalus* | MH016210 | Radchenko et al. 2018 |
| *Megalocottus platycephalus* | MH016211 | Radchenko et al. 2018 |
| *Megalocottus platycephalus* | MH016212 | Radchenko et al. 2018 |
| *Megalocottus platycephalus* | MK936041 | Balakirev et al. 2019 |
| *Megalocottus platycephalus* | MK936042 | Balakirev et al. 2019 |
| *Mesocottus haitej* | KF170219 | Shedko et al. 2013 |
| *Mesocottus haitej* | KF170218 | Shedko et al. 2013 |
| *Microcottus sellaris* | KM057906 | Smith and Busby 2014 |
| *Microcottus sellaris* | LC125750 | Togashi 2019 |
| *Myoxocephalus aenaeus* | KC015709 | McCusker et al. 2013 |
| *Myoxocephalus aenaeus* | KC015707 | McCusker et al. 2013 |
| *Myoxocephalus aenaeus* | MH379082 | Stoeckle et al. 2018 |
| *Myoxocephalus brandtii* | EU200465 | Sharina et al. 2008 |
| *Myoxocephalus brandtii* | KC351874 | Podlesnykh et al. 2013 |
| *Myoxocephalus brandtii* | KC351875 | Podlesnykh et al. 2013 |
| *Myoxocephalus brandtii* | KC351876 | Podlesnykh et al. 2013 |
| *Myoxocephalus brandtii* | EU200463 | Sharina et al. 2016 |
| *Myoxocephalus brandtii* | JF278611 | Podlesnykh et al.2016 |
| *Myoxocephalus brandtii* | MN115337 | Radchenko et al. 2020 |
| *Myoxocephalus jaok* | KC351879 | Podlesnykh et al. 2013 |
| *Myoxocephalus jaok* | KC351880 | Podlesnykh et al. 2013 |
| *Myoxocephalus jaok* | MN115336 | Radchenko et al. 2020 |
| *Myoxocephalus jaok* | MN162695 | Balakirev et al. 2020 |
| *Myoxocephalus jaok* | MN162694 | Balakirev et al. 2020 |
| *Myoxocephalus ochotensis* | JF278618 | Podlesnykh et al.2016 |
| *Myoxocephalus ochotensis* | MH595733 | Petrovskaya et al. 2019 |
| *Myoxocephalus ochotensis* | MN115339 | Radchenko et al. 2020 |
| *Myoxocephalus octodecemspinosus* | KC015729 | McCusker et al. 2013 |
| *Myoxocephalus octodecemspinosus* | KC015728 | McCusker et al. 2013 |
| *Myoxocephalus octodecemspinosus* | KY250721 | Johansson et al. 2017 |
| *Myoxocephalus octodecemspinosus* | MN883270 | Stoeckle et al. 2020 |
| *Myoxocephalus polyacanthocephalus* | HQ712665 | Mecklenburg et al. 2016 |
| *Myoxocephalus polyacanthocephalus* | LC125665 | Togashi et al. 2019 |
| *Myoxocephalus polyacanthocephalus* | MK621914 | Balakirev et al. 2019 |
| *Myoxocephalus polyacanthocephalus* | MK621915 | Balakirev et al. 2019 |
| *Myoxocephalus polyacanthocephalus* | MN115338 | Radchenko et al. 2020 |
| *Myoxocephalus quadricornis* | KJ128649 | Bergsten et al. 2014 |
| *Myoxocephalus quadricornis* | KJ128648 | Bergsten et al. 2014 |
| *Myoxocephalus quadricornis* | MG421784 | Dewaard 2018 |
| *Myoxocephalus quadricornis* | MG422128 | Dewaard 2018 |
| *Myoxocephalus quadricornis* | MG422195 | Dewaard 2018 |
| *Myoxocephalus quadricornis* | MG422697 | Dewaard 2018 |
| *Myoxocephalus quadricornis* | MG422966 | Dewaard 2018 |
| *Myoxocephalus quadricornis* | MG423121 | Dewaard 2018 |
| *Myoxocephalus quadricornis* | MG421040 | Dewaard 2018 |
| *Myoxocephalus quadricornis* | MG421042 | Dewaard 2018 |
| *Myoxocephalus quadricornis* | MG421069 | Dewaard 2018 |
| *Myoxocephalus quadricornis* | MG421145 | Dewaard 2018 |
| *Myoxocephalus quadricornis* | MG421164 | Dewaard 2018 |
| *Myoxocephalus quadricornis* | MG421255 | Dewaard 2018 |
| *Myoxocephalus quadricornis* | MG421302 | Dewaard 2018 |
| *Myoxocephalus quadricornis* | MG422195 | Dewaard 2018 |
| *Myoxocephalus quadricornis* | MG422966 | Dewaard 2018 |
| *Myoxocephalus quadricornis* | MG423121 | Dewaard 2018 |
| *Myoxocephalus quadricornis* | MG421387 | Dewaard 2018 |
| *Myoxocephalus quadricornis* | MG421539 | Dewaard 2018 |
| *Myoxocephalus quadricornis* | MG421699 | Dewaard 2018 |
| *Myoxocephalus quadricornis* | MG422128 | Dewaard 2018 |
| *Myoxocephalus quadricornis* | MG421777 | Dewaard 2018 |
| *Myoxocephalus quadricornis* | MG421784 | Dewaard 2018 |
| *Myoxocephalus quadricornis* | MG422128 | Dewaard 2018 |
| *Myoxocephalus quadricornis* | MG422697 | Dewaard 2018 |
| *Myoxocephalus quadricornis* | MG421040 | Dewaard 2018 |
| *Myoxocephalus quadricornis* | MG421042 | Dewaard 2018 |
| *Myoxocephalus quadricornis* | MG421069 | Dewaard 2018 |
| *Myoxocephalus quadricornis* | MG421145 | Dewaard 2018 |
| *Myoxocephalus quadricornis* | MG421164 | Dewaard 2018 |
| *Myoxocephalus quadricornis* | MG421255 | Dewaard 2018 |
| *Myoxocephalus quadricornis* | MG421302 | Dewaard 2018 |
| *Myoxocephalus scorpioides* | KX586202 | Oliveira et al. 2016 |
| *Myoxocephalus scorpioides* | KX586203 | Oliveira et al. 2016 |
| *Myoxocephalus scorpioides* | KX586204 | Oliveira et al. 2016 |
| *Myoxocephalus scorpioides* | KX586205 | Oliveira et al. 2016 |
| *Myoxocephalus scorpioides* | MG421646 | Dewaard 2018 |
| *Myoxocephalus scorpioides* | MG421807 | Dewaard 2018 |
| *Myoxocephalus scorpioides* | MG422030 | Dewaard 2018 |
| *Myoxocephalus scorpius* | MK321578 | Li et al. 2019 |
| *Myoxocephalus scorpius* | KC015730 | McCusker et al. 2013 |
| *Myoxocephalus scorpius* | KC015732 | McCusker et al. 2013 |
| *Myoxocephalus scorpius* | KC015731 | McCusker et al. 2013 |
| *Myoxocephalus scorpius* | KJ205410 | Knebelsberger et al. 2014 |
| *Myoxocephalus scorpius* | JN312477 | Knebelsberger et al. 2014 |
| *Myoxocephalus scorpius* | LC125700 | Togashi et al. 2019 |
| *Myoxocephalus scorpius* | MK321578 | Li et al. 2019 |
| *Myoxocephalus stelleri* | EU200461 | Sharina et al. 2016 |
| *Myoxocephalus stelleri* | MT258533 | Radchenko et al. 2020 |
| *Myoxocephalus stelleri* | MN115333 | Radchenko et al. 2020 |
| *Myoxocephalus stelleri* | MN115335 | Radchenko et al. 2020 |
| *Myoxocephalus stelleri* | MN115334 | Radchenko et al. 2020 |
| *Myoxocephalus stelleri* | MN115331 | Radchenko et al. 2020 |
| *Myoxocephalus stelleri* | KY062754 | Moreva et al. 2020 |
| *Myoxocephalus thompsonii* | EU524915 | Hubert et al. 2012 |
| *Myoxocephalus thompsonii* | EU524916 | Hubert et al. 2012 |
| *Myoxocephalus thompsonii* | HQ557195 | April et al. 2016 |
| *Myoxocephalus thompsonii* | KX145213 | Schoelinck et al. 2017 |
| *Myoxocephalus thompsonii* | KX145111 | Schoelinck et al. 2017 |
| *Trachidermus fasciatus* | JX017305 | Zeng et al 2012 |
| *Triglopsis quadricornis* | EU524913 | Hubert et al. 2012 |

**References**

April,J.; Mayden,R.L.; Hanner,R.H.;Bernatchez,L. Genetic calibration of species diversity among North America's freshwater fishes.Proc*. Natl. Acad. Sci*. *U.S.A****.* 2011**, *108*, 10602-10607.

Balakirev E.S.; Cherepkova E.V; Kravchenko A. Yu.; Saveliev P.A.; Semenchenko A.A.; Ayala F.J. Complete mitochondrial genome of the Belligerent sculpin *Megalocottus platycephalus* (Cottoidei: Cottidae). *Mitochondrial DNA Part B*, **2019**, *4:2*, 2980-2981.

Balakirev E.S.; Kravchenko A. Yu.; Cherepkova,E.V.; Saveliev P.A.;Semenchenko A.A.; Ayala F.J. Complete mitochondrial genome of the Plain sculpin *Myoxocephalus jaok* (Scorpaeniformes, Cottidae). *Mitochondrial DNA Part B*, **2020**, *5:2*, 1295-1296.

Balakirev,E.S.; Kravchenko,A.Y.; Saveliev,P.A.; Semenchenko,A.A.; Ayala,F.J. Complete mitochondrial genome of the great sculpin *Myoxocephalus polyacanthocephalus* (Cottoidei: Cottidae). *Mitochondrial DNA Part B Res*. **2019,** *4,* 2361-2362.

Bentley, A.C.; Wiley,E.O. University of Kansas Biodiversity Institute barcoding initiative. Unpublished. Uploaded to GenBank on 11/02/2014.

Bergsten, J.; Englund, M.; Ericsson,P. A DNA key to all Swedish vertebrates. Unpublished. Uploaded to GenBank on 16/06/2014.

Buser,T.J.;Andres Lopez,J. Molecular phylogenetics of sculpins of the subfamily Oligocottinae (Cottidae). *Mol. Phylogenet. Evol*. **2015**, *86*, 64-74.

Dewaard,J.R. BIOUG Archive GGBN Data Release. Unpublished. Uploaded to GenBank on 10/112018.

Elz,A.; Schwenke,P.; Park,L.K. NWFSC Forensic Marine Fish Voucher Collection. Unpublished. Uploaded to GenBank on 05/06/2014.

Hubert,N.; Hanner,R.; Holm,E.; Mandrak,N.E.; Taylor,E.; Burridge,M.;Watkinson,D., Dumont,P.; Curry,A.; Bentzen,P.; Zhang,J.; April,J.; Bernatchez,L. Identifying Canadian Freshwater Fishes through DNA Barcodes. *PLoS ONE* **2008**, *3,* E2490.

Johansson,J., Landry,D.; Whittaker,O. BOLD Student Data Portal Data Release. Unpublished. Uploaded to GenBank on 10/05/2017.

Kim, I.H.;Song,Y.S.; Kim,Y.K.;Cang,C.B.; Kim,W.B.;Kim,S.Y. The Complete Mitochondrial Genome of the *Gymnocanthus intermedius* and *G. herzensteini* (Perciformes: Zoarcidae). *Mitochondrial DNA Part B* **2019**, *4*, 2660-2661.

Knebelsberger,T.; Landi,M.;Neumann,H.; Kloppmann,M.; Sell,A.F.; Campbell,P.D.; Laakmann,S.; Raupach,M.J.; Carvalho,G.R.; Costa,F.O. A reliable DNA barcode reference library for the identification of the North European shelf fish fauna*. Mol Ecol Resour.* **2014**, *14*, 1060-1071.

Li, A.; Munehara, H. Complete mitochondrial genome of the Antlered sculpin *Enophrys diceraus* (Scorpaeniformes, Cottidae). *Mitochondrial DNA* **2013**, 26, 125-126.

Li,Y.; Song,P.; Feng,J.; Zhang,N.; Zhang,R.; Lin,L. Complete mitochondrial genome sequence and phylogenetic analysis of *Myoxocephalus scorpius.* *Mitochondrial DNA B Res.* **2019**, 4, 862-863.

McCusker, M.R.; Denti, D.; Van Guelpen, L.; Kenchington, E; Bentzen, P. Barcoding Atlantic Canada's commonly encountered marine fishes. *Mol. Ecol. Resour*. **2013**, *13,* 177-188.

Mecklenburg, C.W.; Moller,P.R.; Steinke,D. Biodiversity of arctic marine fishes: taxonomy and zoogeography. *Mar Biodivers* **2011**, *41*, 109-140.

Moreva,I., Radchenko,O., Petrovskaya,A. and Borisenko,S. Molecular genetic and karyological analysis of antlered sculpins of Enophrys diceraus group (Cottidae). *Russian Journal of Genetics* **2017**, *53*, 1030-1041.

Oliveira,L.M.; Knebelsberger,T.; Landi,M.,; Soares,P.; Raupach,M.J.; Costa,F.O. Assembling and auditing a comprehensive DNA barcode reference library for European marine fishes. *J. Fish Biol.* **2016**, *89*, 2741-2754.

Petrovskaya,A.; Radchenko,O. Molecular-Genetic Differentiation of the Belligerent Sculpin Megalocottus platycephalus (Pallas, 1814) (Scorpaeniformes: Cottidae). *Russian Journal of Marine Biology* **2019**, *45*, 56-66.

Podlesnykh, A.V.; Ryasanova,I.N. Relationships of sculpins (Scorpaeniformes, Cottidae) of Far East Russia from mitochondrial DNA data. Unpublished. Uploaded to GenBank on 25/07/2016.

Radchenko, O.; Petrovskaya, A; Yusupov, R. Differentiation of the far-eastern sculpin *Myoxocephalus stelleri*. Unpublished. Uploaded to GenBank on 04/04/2020.

Ramon,M.L. and Sorenson,M.D. Novel mitochondrial gene rearrangement found in several marine fish. Unpublished. Uploaded to GenBank on 24/06/2016.

Sandel,M.W.; Aguilar,A.; Fast,K.; O'Brien,S.; Lapidus,A.; Allison,D.B.; Teterina,V.; Kirilchik,S. Complete Mitochondrial Genomes of Baikal Oilfishes (Perciformes: Cottoidei), Earth's Deepest-Swimming Freshwater Fishes. *Mitochondrial DNA B Resour* **2017**, *2*, 773-775.

Schoelinck,C., Roy,M., Godbout,V., Castelin,M., Abbott,C. and Gagne,N. From the barcode to the taxonomic revision: a case-study of Canadian freshwater fish barcoding. Unpublished. Uploaded to GenBank on 4/08/2017.

Sharina,S.; Kartavtsev,Y.; Goto,T.; Chichvarkhin,A.; Hanzawa,N.; Sokolovsky,A.; Balanov,A.; Vinnikov,K.;Ivankov,V. Cytochrome oxidase 1 gene sequence analysis in six flatfish species (Teleostei, Pleuronectidae) of Far East Russia with inferences in phylogeny and taxonomy. *Mitochondrial DNA* **2009**, *19*, 479-89.

Shedko,S.V.; Miroshnichenko.I.L.;Nemkova,G.A. Complete mitochondrial genome of the poorly known Amur sculpin *Mesocottus haitej* (Cottoidei: Cottidae). *Mitochondrial DNA* **2013**, *26*, 147-148.

Smith, W.L.; Busby, M.S. Phylogeny and taxonomy of sculpins, sandfishes, and snailfishes (Perciformes: Cottoidei) with comments on the phylogenetic significance of their early-life-history specializations. *Mol. Phylogenet. Evol*. **2014**, *79*, 332–352.

Stoeckle, M.Y.;Das Mishu,M.; Charlop-Powers,Z. GoFish: A versatile nested PCR strategy for environmental DNA assays for marine vertebrates. *PLoS ONE* **2018**, *13*, E0198717.

Swanburg, T.; Horne, J.B.; Baillie,S.; King,S.D.; McBride,M.C.; Mackley,M.P.; Paterson,I.G.; Bradbury,I.R.; Bentzen,P. Complete mitochondrial genomes for *Icelus spatula*, *Aspidophoroides olrikii* and *Leptoclinus maculatus*: pan-Arctic marine fishes from *Canadian waters. Mitochondrial DNA* **2016**, *4*, 2982-2983.

Togashi, K.; Yamazaki, A.; Awata, S.; Koya, Y.; Abe, T.; Tsuruoka, S.; Takeshima, H.; Malkevich, A.; Munehara, H. Molecular phylogeny and Adaptive radiation of Cottoidei. Unpublished. Uploaded to GenBank on 14/02/2019.

Yang,L., Zhu,K. and Fang,J. The complete mitochondrial genome of Cottiusculus nihonkaiensis. Unpublished. Uploaded to GenBank on 12/11/2019.

Zeng,Z.; Liu,Z.Z.;,Pan,L.D.;Tang,S.J.; Wang,C.T.; Tang,W.Q.;Yang,J.Q. Complete mitochondrial genome of the endangered roughskin sculpin *Trachidermus fasciatus* (Scorpaeniformes, Cottidae). *Mitochondrial DNA* **2012**, *23*, 435-437.
