# Supplementary material for: Genetic Evidence for a Mixed Composition of the Genus Myoxocephalus (Cottoidei: Cottidae) Necessitates Generic Realignment
Source: Genes (Basel). 2020 Sep 11;11(9):1071. doi: 10.3390/genes11091071 (PMC7564937; doi:10.3390/genes11091071)
Supplement: Supplementary file 1 [file genes-11-01071-s001.zip › Table_S3_Best_substitution_models.doc]

**Table S3. The best model of substitution under the maximum likelihood criterion**

| Gene | Best model | BIC Score | AIC value | ML value |
| --- | --- | --- | --- | --- |
| *ND1* | TN93+G+I | 12447.296 | 12082.962 | -5995.375 |
| *ND2* | TN93+G+I | 14322.051 | 13954.508 | -6931.155 |
| *COI* | HKY+G+I | 16010.174 | 15632.728 | -7771.300 |
| *COII* | HKY+G | 6891.731 | 6558.624 | -3235.174 |
| *ATP8* | HKY+G | 1874.663 | 1604.387 | -757.625 |
| *ATP6* | HKY+G+I | 8902.247 | 8561.774 | -4235.743 |
| *COIII* | HKY+G+I | 8456.837 | 8110.130 | -4009.939 |
| *ND3* | TN93+G+I | 4623.057 | 4306.136 | -2106.772 |
| *ND4L* | HKY+G | 3376.157 | 3080.313 | -1495.837 |
| *ND4* | GTR+G+I | 17527.627 | 17122.353 | -8512.092 |
| *ND5* | TN93+G+I | 21868.194 | 21474.906 | -10691.396 |
| *ND6* | HKY+G | 6867.721 | 6546.957 | -3229.297 |
| *CytB* | HKY+G | 13870.725 | 13515.225 | -6713.530 |
| *18S* | JC | 5437.625 | 5142.515 | -2536.220 |
| *ITS1* | T92+G | 2497.327 | 2238.848 | -1081.203 |
| *5.8S* | JC | 721.309 | 511.686 | -220.421 |
| *ITS2* | T92+G | 1527.763 | 1280.730 | -602.066 |
| *28S* | TN93+G+I | 11326.356 | 10943.882 | -5429.914 |

Partitioned analysis was performed with PartitionFinder 2.1.1 (Lanfear et al. 2012) that allowed the overall rate to be different across partitions including each protein-coding gene.

**References**

Lanfear, R.; Calcott, B.; Ho, S.Y.; Guindon, S. PartitionFinder: combined selection of partitioning schemes and substitution models for phylogenetic analyses. *Mol. Biol. Evol.* **2012,** 29, 1695–1701.
