# Supplementary material for: Genetic Evidence for a Mixed Composition of the Genus Myoxocephalus (Cottoidei: Cottidae) Necessitates Generic Realignment
Source: Genes (Basel). 2020 Sep 11;11(9):1071. doi: 10.3390/genes11091071 (PMC7564937; doi:10.3390/genes11091071)
Supplement: Supplementary file 1 [file genes-11-01071-s001.zip › Text_S1_rDNA_Values_of_log likelihood.doc]

**Text S1. Values of log likelihood, standard deviations of split frequencies, and convergence diagnostic for the rDNA and *COI* genes**

The complete dataset was also analyzed by Bayesian inference using MrBayes v. 3.2.7a (released March 6, 2019; Ronquist et al. 2012) under the GTR+G+I model. Analyses were performed as two independent runs, each with four incrementally heated Metropolis-coupled Monte-Carlo Markov Chains. Output trees and data were sampled for every 500 generations. Likelihood values reached a plateau within 15,000 – 25,000 generations. A total of 4002 trees in two files were read and 3002 of them were sampled.

For the rDNA sequences the log likelihood values increased from below -17326.723 to around -11392.473 in the first 5,000 generations and then to around -11373.839 after one million generations. The likelihood of the best state for “cold” chain of run 1 was -11357.95 and the likelihood of the best state for “cold” chain of run 2 was -11357.64. The average standard deviation of split frequencies was 0.00123 after 1,000,000 generations indicating stationary conditions. A convergence diagnostic, the Potential Scale Reduction Factor (PSRF) (Gelman and Rubin 1992) was between 1.000 and 1.001 for all parameters, thus, indicating a good sample from the posterior probability distribution.

For the *COI* gene the log likelihood values increased from below -6493.908 to around -3204.399 in the first 5,000 generations and then to around -3172.989 after 500,000 generations. The likelihood of the best state for “cold” chain of run 1 was -3135.22 and the likelihood of the best state for “cold” chain of run 2 was -3135.37. The average standard deviation of split frequencies was 0.009293 after 500,000 generations indicating stationary conditions. A convergence diagnostic was between 1.000 and 1.003 for all parameters.

**References**

Gelman, A.; Rubin, D.B. Inference from iterative simulation using multiple sequences. *Stat. Sci.* **1992**, 7, 457–511.

Ronquist, F.; Teslenko, M.; van der Mark, P.; Ayres, D.; Darling, A.; Höhna, S.; Larget, B.; Liu, L.; Suchard, M.A.; Huelsenbeck, J.P. MrBayes 3.2: Efficient Bayesian phylogenetic inference and model choice across a large model space. *Syst. Biol.* **2012**, 61, 1–4.
